# The association between nutritional adequacy and 28-day mortality in the critically ill is not modified by their baseline nutritional status and disease severity

**DOI:** 10.1186/s13054-019-2500-z

**Published:** 2019-06-18

**Authors:** Charles Chin Han Lew, Gabriel Jun Yung Wong, Ka Po Cheung, Robert J. L. Fraser, Ai Ping Chua, Mary Foong Fong Chong, Michelle Miller

**Affiliations:** 1 Nutrition and Dietetics, College of Nursing and Health Sciences, Flinders University, GPO Box 2100, Adelaide, 5001 South Australia; 2 Dietetics and Nutrition Department, Ng Teng Fong General Hospital, 1 Jurong East Street 21, Singapore, 609606 Singapore; 3 Department of Gastroenterology and Hepatology, College of Medicine and Public Health, Flinders University, GPO Box 2100, Adelaide, 5001 South Australia; 4 Department of Respiratory Medicine, Ng Teng Fong General Hospital, 1 Jurong East Street 21, Singapore, 609606 Singapore; 5 Saw Swee Hock School of Public Health, National University of Singapore, 12 Science Drive 2, #10-01, Singapore, 117549 Singapore

**Keywords:** NUTRIC, Malnutrition, Mortality, Nutritional support, Critical illness

## Abstract

**Background:**

During the initial phase of critical illness, the association between the dose of nutrition support and mortality risk may vary among patients in the intensive care unit (ICU) because the prevalence of malnutrition varies widely (28 to 78%), and not all ICU patients are severely ill. Therefore, we hypothesized that a prognostic model that integrates nutritional status and disease severity could accurately predict mortality risk and classify critically ill patients into low- and high-risk groups. Additionally, in critically ill patients placed on exclusive nutritional support (ENS), we hypothesized that their risk categories could modify the association between dose of nutrition support and mortality risk.

**Methods:**

A prognostic model that predicts 28-day mortality was built from a prospective cohort study of 440 patients. The association between dose of nutrition support and mortality risk was evaluated in a subgroup of 252 mechanically ventilated patients via logistic regressions, stratified by low- and high-risk groups, and days of exclusive nutritional support (ENS) [short-term (≤ 6 days) vs. longer-term (≥ 7 days)]. Only the first 6 days of ENS was evaluated for a fair comparison.

**Results:**

The prognostic model demonstrated good discrimination [AUC 0.78 (95% CI 0.73–0.82), and a bias-corrected calibration curve suggested fair accuracy. In high-risk patients with short-term ENS (≤ 6 days), each 10% increase in goal energy and protein intake was associated with an increased adjusted odds (95% CI) of 28-day mortality [1.60 (1.19–2.15) and 1.47 (1.12–1.86), respectively]. In contrast, each 10% increase in goal protein intake during the first 6 days of ENS in high-risk patients with longer-term ENS (≥ 7 days) was associated with a lower adjusted odds of 28-day mortality [0.75 (0.57–0.99)]. Despite the opposing associations, the mean predicted mortality risks and prevalence of malnutrition between short- and longer-term ENS patients were similar.

**Conclusions:**

Combining baseline nutritional status and disease severity in a prognostic model could accurately predict 28-day mortality. However, the association between the dose of nutrition support during the first 6 days of ENS and 28-day mortality was independent of baseline disease severity and nutritional status.

**Electronic supplementary material:**

The online version of this article (10.1186/s13054-019-2500-z) contains supplementary material, which is available to authorized users.

## Background

The optimal daily amounts of energy and protein that result in a lower mortality risk in critically ill patients remain uncertain. Heyland et al. [1] proposed that nutritional support may not benefit all patients and consequently developed the Nutrition Risk in Critically Ill score (NUTRIC) to identify patients who would derive the most benefit from nutritional support. This score was subsequently modified to exclude interleukin-6 [modified-NUTRIC (mNUTRIC)] and currently comprises variables such as age, Acute Physiology and Chronic Health Evaluation II score, Sequential Organ Failure Assessment (SOFA) score, length of hospitalization before admission to the intensive care unit (ICU), and number of comorbidities [2]. None of these is nutritional parameters, and it is arguable that the mNUTRIC is a disease severity score. This concept is supported by data from a study by Lew et al. [3] wherein a poor concordance was demonstrated between the mNUTRIC score and the Subjective Global Assessment (SGA)—a validated nutritional assessment tool that has strong mortality prognostic value in critically ill patients [4, 5].

The mNUTRIC has a maximum score of 9, in which the scores 0 to 4 and 5 to 9 are classified as low-mNUTRIC and high-mNUTRIC, respectively. Adequate energy and protein intakes were observed to benefit only high-mNUTRIC patients, with no effect on low-mNUTRIC patients [2, 6, 7]. However, several recent studies have reported conflicting results [8, 9]. Lew et al. [10] recently validated the mNUTRIC score and observed that the association between mNUTRIC score and 28-day mortality was modified by the timing and dose of nutritional support [10]. Specifically, the study suggested that early high energy and protein intakes were associated with a higher risk of 28-day mortality in high-mNUTRIC patients with short-term nutritional support (≤ 6 days), whereas the inverse was observed in those with longer-term nutritional support (≥ 7 days) [10]. However, the median mNUTRIC scores of these two groups of patients (receiving ≤ 6 days vs. ≥ 7 days of nutritional support) at ICU admission were similar, which suggested that the associations between early high energy and protein intakes and 28-day mortality were not modified by baseline disease severity measured by the mNUTRIC score.

It is unclear if the above results reflect the absence of nutritional parameters in the mNUTRIC score because intuitively, malnourished patients would be expected to require more energy and protein to overcome the deleterious effects of critical illness. Lew et al. [3] previously demonstrated that disease severity (measured by the mNUTRIC) and nutritional status (measured by the SGA) independently and in combination can predict mortality [3, 4]. A recent review also recommends simultaneous use of both the mNUTRIC and SGA for complete nutritional evaluation for the critically ill [11]. In light of this, we aimed to develop a new prognostic model, namely the Global Index of Mortality Probability in the Severely ill (GLIMPSE) that combines both mNUTRIC and 7-point SGA to classify patients into low- and high-risk of 28-day mortality (low-GLIMPSE and high-GLIMPSE, respectively). In addition, we evaluated whether GLIMPSE categories could modify the association between dose of nutrition support during the first 6 days of exclusive nutritional support (ENS) and 28-day mortality in a single-centre cohort study.

## Methods

### Setting and patients

This was a prospective observational cohort study conducted in the ICU (35 beds) of Ng Teng Fong General Hospital (Singapore). The ICU functions as a closed unit, in which board-certified intensivists and residents provide care for medical, surgical, trauma, cardiac, and neurological patients. The intensivists and nurses were blinded to the objective of the study. Patients are classified as “critically ill” if they are mechanically ventilated and require the support of two or more organ systems. They are downgraded to high dependency status once they are extubated from mechanical ventilation.

To develop GLIMPSE, consecutive patients admitted between August 2015 and October 2016 were screened for enrolment. Patients who were at least 21 years old, had been admitted to the ICU at least 24 h prior to the screening, and whose nutritional status was established within 48 h were enrolled. Nutritional status was established by the 7-point SGA, and details have been previously published [4]. Briefly, each 1 point decrease in the 7-point SGA (indicative of a greater degree of malnutrition) was associated with a higher risk of 28-day mortality [4]. Patients who were readmitted to the ICU within the same hospitalization were excluded.

The modifying effects of GLIMPSE categories on the association between the dose of nutrition support during the first 6 days of ENS and 28-day mortality were evaluated in a subgroup of patients. These patients had experienced at least 48 h of mechanical ventilation and were not pronounced moribund (had medical orders not to resuscitate or had poor prognosis) within 48 h of ICU admission.

The Domain Specific Review Board approved this study (NHG DSRB Ref: 2014/00878), and informed consent was not required because no attempt was made to change the standard of care in the study.

### Data collection

Patient demographics, admission diagnoses, adequacy of exclusive nutritional support (ENS), and mortality outcome were prospectively recorded in the electronic medical records and retrieved. Since mNUTRIC was not part of routine care, it was calculated at the end of the study. Details of the collection of energy and protein intakes via ENS have been previously published [10]. Briefly, adequacy of nutritional support was calculated by dividing the total enteral and/or parenteral nutrition (energy and protein) intake as well as energy provided by propofol and intravenous dextrose by a number of days on ENS and expressed as a percentage of the goals established at ICU admission. This information was recorded from ICU admission to a maximum of 14 days, unless death occurred earlier.

### Development of GLIMPSE

Variables demonstrated to be associated with mortality outcomes in our previous studies were included in GLIMPSE. These comprised the following: (1) disease severity, as measured by the mNUTRIC score [3]; (2) nutritional status, as measured by the 7-point SGA [4]; and (3) cardiopulmonary resuscitation before ICU admission [4]. As there were three predictors, it was estimated that the model required at least 74 events (deaths) in order to ensure model stability [12]. The three predictors were fitted into a multivariable logistic model to predict 28-day mortality. This generated weighted coefficients and a constant that could be used to calculate the predicted mortality risk of patients. More importantly, a logistic model is required for the measurement of internal validity. The internal validity of GLIMPSE was assessed via a bootstrapping technique, in which 1000 re-samples of the entire cohort were created to quantify the discrimination and calibration accuracy of GLIMPSE (R package version 3.5.1. http://CRAN.R-project.org/package=rms). Discrimination was assessed by using the *C*-index (adjusted for optimism via bootstrapping). Calibration was assessed graphically by preparing a bias-corrected calibration curve and assessing its slope.

### Validity of GLIMPSE as an assessment tool for nutritional support

Lew et al. [10] showed that the associations between early high energy and protein intakes and 28-day mortality in high-mNUTRIC patients with ≤ 6 days and ≥ 7 days of ENS were different (increased mortality risk vs. reduced mortality risk, respectively) and the mNUTRIC of these two groups of patients were similar [10]. We adopted the same method used in the earlier study to determine the validity of GLIMPSE in the same subgroup of patients. First, the mortality risks of patients predicted by GLIMPSE were stratified into low- and high-GLIMPSE by using the Youden Index [13]. Second, multivariable logistic regressions were used to determine the associations between energy and protein adequacies and 28-day mortality, stratified by GLIMPSE risk groups and the duration of ENS exposure (≤ 6 days and ≥ 7 days). To avoid multicollinearity, covariate adjustment was not carried out on variables used to calculate the mNUTRIC score [6]. Statistical analyses were performed using STATA 14.2 (Stata Corp., College Station, TX, USA) and R package (version 3.5.1). For all comparisons, interactions, and associations, a *p* value < 0.05 was considered statistically significant.

## Results

**Fig. 1:**
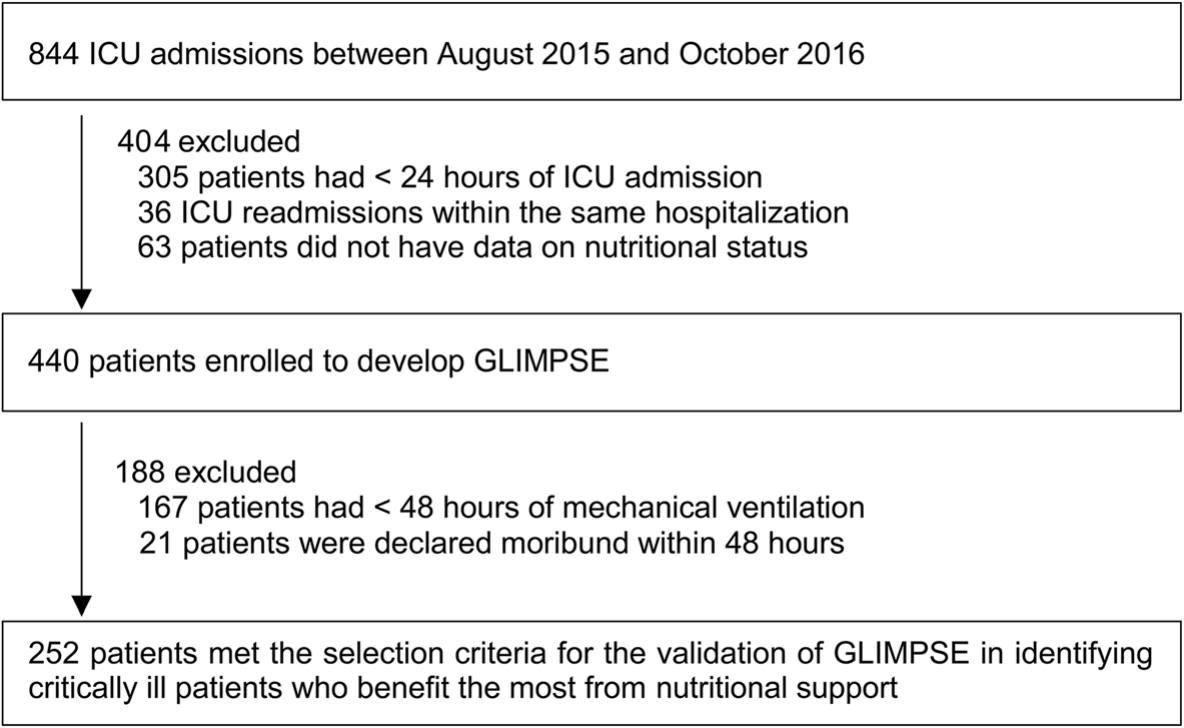
Enrolment of patients

There were 440 patients enrolled, and no patients were lost to follow-up (Fig. 1). Mortality at day 28 following ICU admission was 28.0%. Survivors had lower disease severity and were more likely to be well-nourished compared to nonsurvivors (Table 1). Data from a subgroup of 252 patients were used to evaluate the modifying effects of GLIMPSE categories on the association between the dose of energy and protein intakes during the first 6 days of ENS and 28-day mortality. Patients who were excluded had similar characteristics to those included in the validation group apart from a lower SOFA score (mean, 8.1 vs. 9.2, *p* < 0.001) and a higher number of comorbidities (median, 3 vs. 2, *p* < 0.001). The characteristics of the 252 patients were stratified by their duration of exposure to ENS (short- vs. longer-term ENS). The characteristics of patients with short- and longer-term ENS were similar except for the type of admission, number of comorbidities, and admission diagnosis.

**Table 1:** Characteristics of the enrolled patients

Patient characteristics	Enrolled patients (*n* = 440)	Survivors (*n* = 317)	Nonsurvivors (*n* = 123)	*p* value	Short-term ENS (≤ 6 days) (*n* = 106)	Longer-term ENS (≥ 7 days) (*n* = 146)	*p* value
Age (years)	61.4 (15.7)	59.3 (15.9)	66.8 (14.1)	< 0.001	60.4 (16.9)	59.5 (15.5)	0.655
Male	259 [58.9]	192 [60.6]	67 [54.5]	0.244	63 [59.4]	92 [63.0]	0.564
BMI (kg/m^2^)	24.4 [21.3, 28.2]	24.4 [21.2, 28.0]	24.2 [21.3, 28.6]	0.955	24.5 [21.2, 29.5]	24.6 [21.8, 28.4]	0.726
Location before adm.				0.003			0.976
ED/HD/OT	357 [81.1]	268 [84.5]	89 [72.4]		88 [83.0]	121 [82.9]	
Wards	83 [18.9]	49 [15.5]	34 [27.6]		18 [17.0]	25 [17.1]	
Type of adm.				0.006			0.001
Medical	293 [66.6]	199 [62.8]	94 [76.4]		80 [75.5]	79 [54.1]	
Surgery	147 [33.4]	118 [37.2]	29 [23.6]		26 [24.5]	67 [45.9]	
Number of comorbidities	2.0 [1.0, 4.0]	2.0 [1.0, 4.0]	3.0 [2.0, 4.0]	0.069	3.0 [1.0, 4.0]	2.0 [1.0, 3.0]	0.002
LOS before ICU adm. (days)	0.0 [0.0, 1.0]	0.0 [0.0, 1.0]	1.0 [0.0, 3.0]	0.009	0.0 [0.0, 1.3]	0.0 [0.0, 1.3]	0.730
APACHE II	24.5 (8.1)	22.6 (7.4)	29.5 (7.7)	< 0.001	25.4 (8.4)	24.7 (7.6)	0.543
SOFA	8.7 (3.8)	7.9 (3.5)	10.7 (3.8)	< 0.001	9.2 (3.7)	9.1 (3.8)	0.909
mNUTRIC	5.3 (2.1)	4.8 (2.1)	6.6 (1.5)	< 0.001	5.5 (2.3)	5.3 (2.0)	0.531
mNUTRIC ≥ 5	299 [68.0]	187 [59.0]	112 [91.1]	< 0.001	74 [69.8]	100 [68.5]	0.823
Malnutrition	123 [28.0]	72 [22.7]	51 [41.5]	< 0.001	31 [29.2]	35 [24.0]	0.347
Admission reasons				< 0.001			0.020
Cardiovascular	82 [18.6]	43 [13.6]	39 [31.7]		19 [17.9]	18 [12.3]	
Respiratory	84 [19.1]	68 [21.5]	16 [13.0]		24 [22.6]	21 [14.4]	
Sepsis	105 [23.9]	72 [22.7]	33 [26.8]		36 [34.0]	39 [26.7]	
Trauma	12 [2.7]	12 [3.8]	0 [0.0]		4 [3.3]	4 [2.7]	
Metabolic/renal	8 [1.8]	8 [2.5]	0 [0.0]		2 [1.9]	2 [1.4]	
Gastrointestinal	42 [9.6]	29 [9.2]	13 [10.6]		3 [2.8]	8 [5.5]	
Postoperation	13 [3.0]	12 [3.8]	1 [0.8]		4 [3.8]	3 [2.1]	
Orthopaedics	7 [1.6]	6 [1.9]	1 [0.8]		1 [0.9]	2 [1.4]	
Neurological	87 [19.8]	67 [21.1]	20 [16.3]		13 [12.3]	49 [33.6]	
CPR before ICU adm.	53 [12.1]	17 [5.4]	36 [29.3]	< 0.001	18 [17.0]	18 [12.3]	0.297
Length of MV (days)	2.0 [1.0, 5.0]	2.0 [1.0, 4.0]	3.0 [2.0, 6.0]	< 0.001	3.0 [2.0, 4.0]	7.0 [4.0, 13.0]	< 0.001
ICU LOS (days)	2.0 [2.0, 5.0]	2.0 [1.0, 4.0]	3.0 [2.0, 6.0]	0.001	3.0 [2.0, 4.0]	7.0 [4.0, 12.0]	< 0.001
Hospital LOS (days)	14.0 [7.0, 25.0]	15.0 [9.0, 33.0]	9.0 [4.0, 16.0]	< 0.001	8.0 [4.0, 15.3]	24.0 [16.0, 45.0]	< 0.001

Values are mean (standard deviation), median [Q1, Q3], or count [percentage]

*Adm.* admission, *APACHE II* Acute Physiology and Chronic Health Evaluation II, *BMI* body mass index, *CPR* cardiopulmonary resuscitation, *ED* emergency department, *ENS* exclusive nutritional support, *HD* high dependency, *ICU* intensive care unit, *LOS* length of stay, *MV* mechanical ventilation, *mNUTRIC* Modified Nutrition Risk in Critically Ill, *OT* operation theatre, *SOFA* Sequential Organ Failure Assessment

The mean (SD) percentages of energy and protein relative to the requirements for the first 6 days of ICU admission in the validation group were 60.0% (23.6%) [15.2 (6.5) kcal/kg] and 55.1% (24.5%) [0.64 (0.31) g/kg], respectively. The actual energy and protein intakes are summarized in Table 2. During the first 6 days of ENS, patients with longer-term ENS had significantly higher energy and protein intakes than those of the patients with short-term ENS (*p* value < 0.001 for both energy and protein). When stratified by nutritional status, the mean percentages of goal energy and protein intakes between the well-nourished and malnourished patients during the first 6 days of ENS (energy, 63.4% vs. 58.8%, *p* = 0.172; protein, 57.4% vs. 54.2%, *p* = 0.368, respectively) were not signficantly different.

**Table 2:** Comparison of goal and achieved energy and protein intakes between 28-day survivors and nonsurvivors stratified by days of exclusive nutritional support

Nutritional parameters	All patients	Survivors	Nonsurvivors	*p* value
Short-term exclusive nutritional support (≤ 6 days)				
Energy				
Goal (kcal/kg)	25.7 (5.8)	25.5 (5.5)	25.9 (6.3)	0.680
Actual intake (kcal/kg)	12.0 (6.6)	10.0 (6.0)	15.0 (6.4)	< 0.001
Actual intake (% goal/kg)	48.0 (24.9)	40.0 (22.4)	59.2 (24.1)	< 0.001
Protein				
Goal (g/kg)	1.14 (0.23)	1.14 (0.20)	1.15 (0.26)	0.779
Actual intake (g/kg)	0.47 (0.29)	0.39 (0.26)	0.57 (0.30)	0.001
Actual intake (% goal/kg)	41.6 (25.0)	34.7 (21.6)	51.4 (26.4)	0.001
Longer-term exclusive nutritional support (≥ 7 days)				
Energy				
Goal (kcal/kg)	25.6 (4.4)	25.9 (4.4)	24.8 (4.3)	0.166
Actual intake (kcal/kg)	17.5 (5.3)	17.7 (5.2)	16.9 (5.7)	0.389
Actual intake (% goal/kg)	68.7 (18.2)	68.8 (17.8)	68.2 (19.5)	0.857
Protein				
Goal (g/kg)	1.19 (0.22)	1.20 (0.21)	1.15 (0.26)	0.281
Actual intake (g/kg)	0.77 (0.25)	0.79 (0.24)	0.71 (0.28)	0.125
Actual intake (% goal/kg)	64.8 (19.1)	66.1 (18.9)	61.6 (19.4)	0.206

### Development of GLIMPSE

**Fig. 2:**
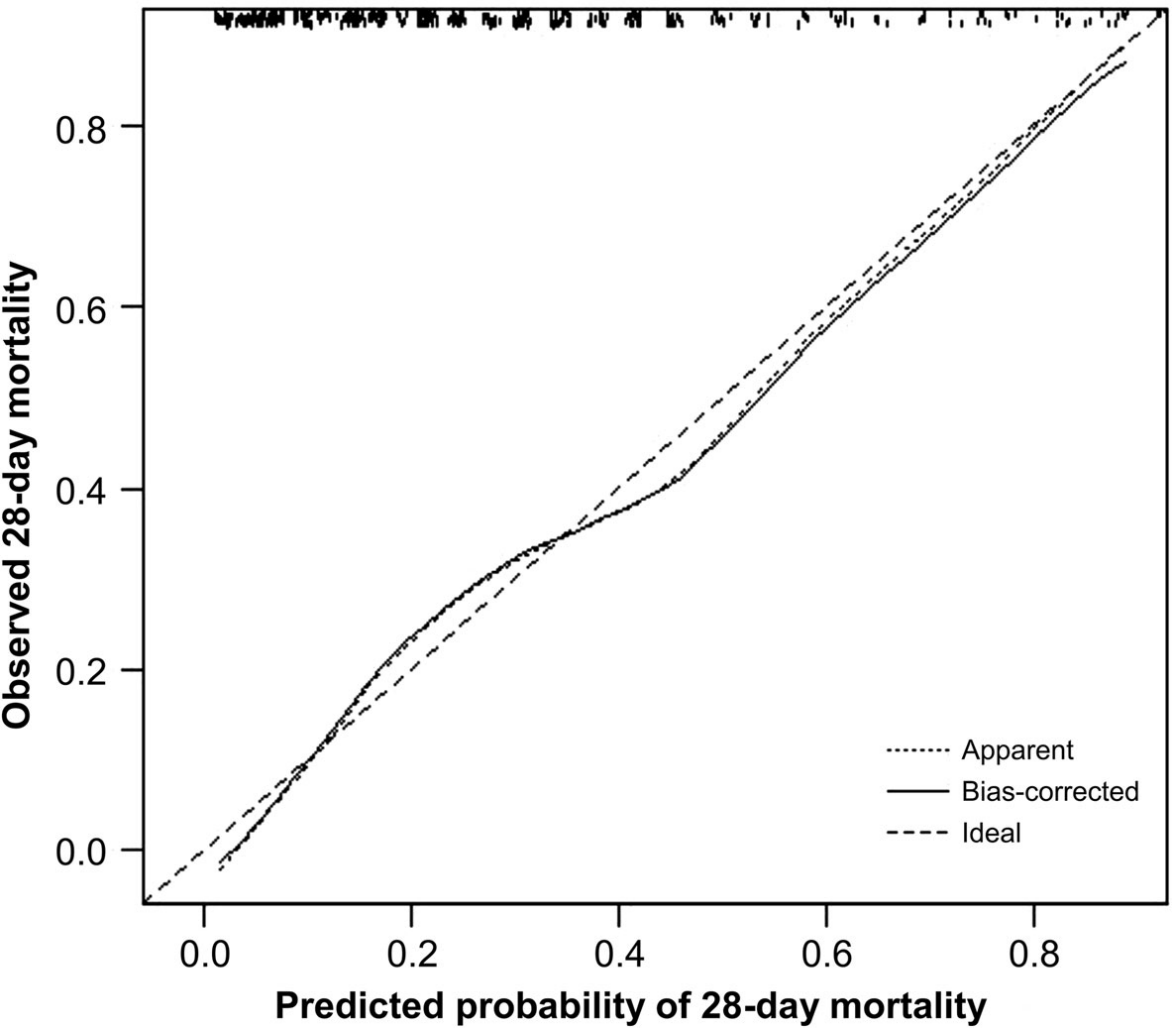
Bias-corrected calibration curve for the prediction of 28-day mortality

The mNUTRIC score, 7-point SGA, and exposure to cardiopulmonary resuscitation before ICU admission were fitted into a multivariable logistic regression, and they were significantly associated with 28-day mortality (refer to Additional file 1 for regression parameters). The resultant equation used to calculate the predicted 28-day mortality risk was − 3.003 + (mNUTRIC × 0.477) + (7-point SGA × − 0.166) + (exposure to cardiopulmonary resuscitation before ICU admission × 1.933). Internal validation in the model via 1000 bootstrap replicates revealed an adjusted *C*-index and max-rescaled *R*^2^ of 0.78 (95% CI 0.73–0.82) and 0.30, respectively. The bias-corrected calibration curve (Fig. 2) suggested overall fair calibration accuracy (slope, 0.98).

### Validity of GLIMPSE as an assessment tool for nutritional support

The optimal cutoff point for defining low- and high-GLIMPSE was 20%, and the *C*-index, sensitivity, and specificity were 0.71, 87.8%, and 54.6%, respectively. The characteristics of patients classified as low- and high-GLIMPSE on the first day of ICU admission were all significantly different except for sex and body mass index (Table 3). However, the mean predicted mortality risks (standard deviation) in patients with short- and longer-term ENS were similar [32.2% (24.1%) vs. 27.8% (21.3%), respectively, *p* = 0.122].

**Table 3:** Characteristics of low- and high-GLIMPSE patients

Patient characteristics	Low-GLIMPSE (*n* = 188)	High-GLIMPSE (*n* = 252)	*p* value
Age (years)	53.6 (16.0)	67.2 (12.8)	< 0.001
Male	115 [61.2]	144 [57.1]	0.396
BMI (kg/m^2^)	25.5 (6.3)	24.9 (5.8)	0.262
Location before adm.			< 0.001
ED/HD/OT	169 [89.9]	188 [74.6]	
Wards	19 [10.1]	64 [25.4]	
Type of adm.			< 0.001
Medical	104 [55.3]	189 [75.0]	
Surgery	84 [44.7]	63 [25.0]	
Number of comorbidities	2.0 [1.0, 3.0]	3.0 [2.0, 4.0]	< 0.001
LOS before ICU adm. (days)	0.0 [0.0. 1.0]	1.0 [0.0, 2.5]	< 0.001
APACHE II	18.6 (5.2)	29.0 (6.9)	< 0.001
SOFA	6.4 (3.0)	10.3 (3.4)	< 0.001
mNUTRIC	3.4 (1.5)	6.7 (1.2)	< 0.001
mNUTRIC ≥ 5 (high-mNUTRIC)	55 [29.3]	244 [96.8]	< 0.001
Malnutrition	29 [15.4]	94 [37.3]	< 0.001
Predicted mortality risk	9.7 (5.0)	41.6 (19.5)	< 0.001
Admission reasons			< 0.001
Cardiovascular	22 [11.7]	60 [23.8]	
Respiratory	39 [20.7]	45 [17.9]	
Sepsis	26 [13.8]	79 [31.4]	
Trauma	11 [5.9]	1 [0.4]	
Metabolic/renal	3 [1.6]	5 [2.0]	
Gastrointestinal	15 [8.0]	27 [10.7]	
Postoperation	9 [4.8]	4 [1.6]	
Orthopaedics	3 [1.6]	4 [1.6]	
Neurological	60 [31.9]	27 [10.7]	
CPR before ICU adm.	1 [0.5]	52 [20.6]	< 0.001
Length of MV (days)	2.0 [1.0, 4.0]	2.0 [1.0, 5.0]	0.039
ICU LOS (days)	2.0 [2.0, 5.0]	3.0 [2.0, 5.0]	0.381
Hospital LOS (days)	13.5 [7.0, 27.0]	14.0 [7.0, 24.0]	0.953

Values are mean (standard deviation), median [Q1, Q3], or count [percentage]

*Adm.* admission, *APACHE II* Acute Physiology and Chronic Health Evaluation II, *BMI* body mass index, *CPR* cardiopulmonary resuscitation, *ED* emergency department, *ENS* exclusive nutritional support, *HD* high dependency, *ICU* intensive care unit, *LOS* length of stay, *MV* mechanical ventilation, *mNUTRIC* Modified Nutrition Risk in Critically Ill, *OT* operation theatre, *SOFA* Sequential Organ Failure Assessment

Several covariates were not adjusted in the multivariable logistic model. Firstly, *location before ICU admission* and *types of admission* were not adjusted although they were significantly associated with mortality at the univariate level (Table 1). When included in a multivariable model (along with GLIMPSE group, adequacy of energy and protein intake, and duration of ENS), these covariates were not significantly associated with mortality (refer to Additional file 1). Secondly, since the *duration of ENS* was correlated with the *duration of mechanical ventilation* (r = 0.82) and *ICU length of stay* (*r* = 0.67), only the duration of ENS was included in the multivariable logistic model [14]. Lastly, time to initiation of enteral feeding was not adjusted because our ICU nutrition policy requires patients to receive enteral nutrition support within 48 h of ICU admission. Therefore, only 12 patients were not enterally fed within 48 h of ICU admission. This resulted in an insignificant association with mortality (*p* = 0.416).

In patients with short-term ENS, the associations between energy and protein intakes and 28-day mortality during the first 6 days of ENS were examined in multivariable logistic regression models (Table 4). There were no interactions between the GLIMPSE groups and energy and protein intakes. Generally, there was a positive association between energy and protein intakes and 28-day mortality in both GLIMPSE groups, but it was statistically significant for patients with high-GLIMPSE only.

**Table 4:** Association between energy and protein intakes and 28-day mortality as well as nutritional status stratified by days on exclusive nutritional support and GLIMPSE groups

Parameters	Short-term ENS (≤ 6 days)		Longer-term ENS (≥ 7 days)		*p* value	
	Low-GLIMPSE* (*n* = 36)	High-GLIMPSE* (*n* = 70)	Low-GLIMPSE* (*n* = 64)	High-GLIMPSE* (*n* = 82)		
Energy intake					0.793^†^	0.035^‡^
Each 10% of goal	1.24 (0.70–2.20)	1.60 (1.19–2.15)	2.17 (0.75–6.26)	0.83 (0.64–1.12)		
	*p* = 0.462	*p* = 0.002	*p* = 0.150	*p* = 0.228		
	GOF = 0.682	GOF = 0.823	GOF = 0.287	GOF = 0.615		
Protein intake					0.904^§^	0.025**
Each 10% of goal	1.20 (0.68–2.11)	1.47 (1.12–1.86)	1.23 (0.73–2.08)	0.75 (0.57–0.99)		
	*p* = 0.535	*p* = 0.004	*p* = 0.428	*p* = 0.043		
	GOF = 0.685	GOF = 0.530	GOF = 0.455	GOF = 0.335		
7-point SGA-categories					0.480^††^	0.633^‡‡^
Well-nourished						
SGA-7	22 [61.1]	34 [48.6]	41 [64.1]	34 [41.5]		
SGA-6	8 [22.2]	11 [15.7]	18 [28.1]	18 [22.0]		
Mildly to moderately malnourished						
SGA-5	3 [8.3]	8 [11.4]	4 [6.3]	14 [17.1]		
SGA-4	2 [5.6]	7 [10.0]	1 [1.6]	9 [11.0]		
SGA-3	1 [2.8]	5 [7.1]	0 [0.0]	5 [6.1]		
Severely malnourished						
SGA-2		4 [5.7]		2 [2.4]		
SGA-1		1 [1.4]		0 [0.0]		

Values are adjusted odds ratio (95% confidence interval) adjusted for days on exclusive nutritional support and count [percentage]. Refer to Additional file 1 for beta weights of potential confounders not included in the model

*ENS* exclusive nutritional support, *GLIMPSE* Global Index of Mortality Probability in the Severely ill, *GOF* goodness of fit derived from Hosmer-Lemeshow test, *SGA* Subjective Global Assessment

*Low- and high-GLIMPSE is defined as the predicted mortality risk of < 20% and > 20%, respectively

^†^Interaction between energy intake and GLIMPSE categories in patients with short-term exclusive nutritional support

^‡^Interaction between energy intake and GLIMPSE categories in patients with longer-term exclusive nutritional support

^§^Interaction between protein intake and GLIMPSE categories in patients with shorter-term exclusive nutritional support

**Interaction between protein intake and GLIMPSE categories in patients with longer-term exclusive nutritional support

^††^Comparison of 7-point SGA categories between low-GLIMPSE patients with short-term and longer-term ENS

^‡‡^Comparison of 7-point SGA categories between high-GLIMPSE patients with short-term and longer-term ENS

Similarly, the association between energy and protein intakes and 28-day mortality during the first 6 days of ENS was examined in patients with longer-term ENS (Table 4). There were significant interactions between the GLIMPSE groups and energy and protein intakes. The associations between energy and protein intakes and 28-day mortality in patients with low- and high-GLIMPSE appeared to be opposite of each other, but only protein intake was significantly associated with 28-day mortality because each 10% increase in protein adequacy was associated with a 25% reduction in the odds of 28-day mortality.

## Discussion

A new prognostic model (GLIMPSE) was developed by combining baseline nutritional status (measured by the 7-point SGA) and disease severity (measured by the mNUTRIC score) to predict 28-day mortality. Internal validation suggested that GLIMPSE had good discrimination and calibration accuracy and was able to identify patients at low and high risk of 28-day mortality. However, the mortality risk established by GLIMPSE did not explain the lack of association between the dose of nutrition support during the first 6 days of ENS and 28-day mortality.

### Prognostic performance

The GLIMPSE model can be viewed as an extension of the mNUTRIC score. The latter had a *C*-index and max-rescaled *R*^2^ of 0.65 and 0.57, respectively [2]. However, subsequent large multicentre cohort studies (*n* > 1000) that sought to validate mNUTRIC demonstrated reduced prognostic performance, with the *C*-index and max-rescaled *R*^2^ ranging from 0.65 to 0.7 and from 0.09 to 0.16, respectively [6, 15]. In our study, the GLIMPSE model demonstrated better prognostic performance than that of the mNUTRIC. This may be attributed to the inclusion of strong independent predictors of mortality (nutritional status and CPR) [4]. Despite its good prognostic performance, the GLIMPSE model was unable to explain the lack of association between early high energy and protein intakes and 28-day mortality.

### Validity of GLIMPSE as an assessment tool for nutritional support

The prevalence of malnutrition in the ICU reportedly ranges from 28 to 78% [4]. Urgent nutritional support, which often translates to early high energy and protein intakes, is recommended for malnourished critically ill patients because it confers clinical benefits [16, 17]. To our knowledge, this has not been examined in randomized controlled trials performed in ICUs because this subgroup of patients is often excluded for ethical reasons. When examined in the subgroup or post hoc analyses, patients at risk of malnutrition did not benefit from higher energy and protein intakes [18, 19]. Given the paucity of evidence, our prospective observational study may help in bridging this knowledge gap, i.e. whether the associations between the dose of energy and protein intakes and mortality risk could be modified by the baseline nutritional status of critically ill patients.

The GLIMPSE tool was developed because it was hypothesized that baseline nutritional status and disease severity could modify the association between the dose of energy and protein intakes and mortality risk. However, this was not supported by the data in the current study. The disparity between early high energy and protein intakes and 28-day mortality in patients with short- and longer-term ENS was unexpected, as was the lack of discrimination by the integration of nutritional status with disease severity in the GLIMPSE model. Further stratification of nutritional status in these two groups of patients also revealed that the associations between energy and protein intakes and 28-day mortality were independent of baseline nutritional status (Table 4). The only overt differences between patients with short- and longer-term ENS were their exposure to surgery and the type of admission diagnosis, with patients who had short-term ENS being more likely to be admitted for medical reasons and having a higher incidence of cardiovascular or respiratory issues. In contrast, a higher proportion of patients with longer-term ENS were admitted with neurological diagnoses (Table 1).

One of the possible explanations for the results of our study could be the metabolic state (i.e. catabolism vs. anabolism) at the initial phase of nutritional support, since this will dictate the fate of exogenous macronutrient metabolism. *Catabolism*—During the acute phase of critical illness, raised concentrations of glucagon, catecholamines, and cortisol may result in anabolic resistance [20]. Therefore, energy and protein provided at this stage may not be effectively used for anabolism or attenuation of catabolism (processes required to treat malnutrition) [21–23]. This has been reported in recent studies, where early high protein intake during the acute phase of critical illness led to increased ureagenesis (reflecting inefficiency in protein metabolism [24–27]). Furthermore, early high energy and protein intakes were associated with poorer clinical outcomes, such as prolonged ICU length of stay and increased risk of infection [18] as well as a greater degree of muscle and functional loss [22, 28, 29]. Mechanistically, these results may be attributed to hindered autophagy [28, 30], attenuation of the nonthyroidal illness syndrome [31] or intramuscular inflammation, and altered hypoxic signalling, which consequently reduces the bioavailability of the intramuscular adenosine triphosphate needed for muscle protein synthesis [29]. *Anabolism*—By contrast, during the later phase of critical illness, inflammation and the levels of hormones responsible for catabolism gradually abate; this may result in more efficient use of energy and protein for anabolism and recovery [32].

The number, type, and degree of insults for each critically ill patient differ widely in the ICU. Therefore, some patients will be in a prolonged catabolic state, whereas others progress quickly into the anabolic phase (the later stage of a critical illness) [32]. From the literature, it may be deduced that the acute state can be as short as 3 days [33] or as long as 7 days [34–36]. In the acute state, high energy and protein intakes have been shown to worsen clinical outcomes, whereas the same nutrient delivery at the later stage has shown to have clinical benefits [33–36]. Variability in the duration of the acute state could explain the different effects of early high energy and protein intakes in our study. That is, the positive association between energy and protein intakes and 28-day mortality in high-GLIMPSE patients with short-term ENS may reflect the consequence of early high energy and protein intakes in an overt state of catabolism, whereas the negative association in patients with longer-term ENS suggests an early resolution of catabolism, and hence, energy and protein could be efficiently used for anabolism. This hypothesis could also explain why Compher et al. [6] observed an inverse association between the dose of nutrition support and mortality in patients with > 4 days of ENS - an observation congruent with patients with longer-term ENS (≥ 7 days) in our study. The inclusion criteria used by Compher et al. [6] could inherently have selected patients who were somewhat in the anabolic phase and hence benefited from higher energy and protein intakes. Therefore, their findings [6] may only apply to patients with > 4 days of ENS, and the extrapolation of these results to recommend early aggressive nutritional support to all severely ill ICU patients may require caution.

The current study had a number of strengths. These include consecutive recruitment and complete follow-up, which minimised the selection and attrition biases. In addition, we avoided an erroneous association between minimal nutritional intake and mortality by excluding moribund patients, since these patients inherently have a minimal nutritional intake, and death reflects disease severity. However, there were several limitations. First, although GLIMPSE was found to have good mortality prognostic performance, it was unable to characterize the dynamic disease progression of critically ill patients. For example, patients admitted with diabetic ketoacidosis and severe pancreatitis may have identical GLIMPSE scores, but the former would have a rapid metabolic recovery, whereas the insult suffered by the latter may worsen. Since GLIMPSE, along with other prognostic scores, such as the mNUTRIC, are measured once at day 2 of ICU admission, they are unable to account for the differing pathophysiologies and disease progressions under different conditions. Therefore, future studies should collect covariates such as levels of organ support and sequential SOFA score to adjust for these time-dependent parameters. Second, the prognostic accuracies of GLIMPSE were based on a single centre with relatively small sample size, and larger multi-site studies are required to more accurately estimate the prognostic performance of the GLIMPSE model. Third, as in all observational studies, the possibility of residual confounding factors limited the interpretation of the results in this study to the association rather than to the causality. Lastly, nutritional status was not reassessed during the course of ICU admission. Therefore, some patients who were well-nourished at baseline may have become malnourished during their stay in the ICU.

### Future research and clinical implications

As the fate of nutrient metabolism during the acute and later phases of critical illness is different, it is crucial for future studies to identify the biomarkers that can accurately define the duration of the different phases to better guide nutritional support in the ICU [32]. Before this is achieved, perhaps the timing and dose of energy and protein intakes should be guided by the surrogates of inflammation resolution, e.g. down-trending of high-sensitivity C-reactive protein, reduced insulin resistance (improved glycaemic control in patients without diabetes [37]), or improvement in organ functions (down-trending of the sequential SOFA scores) [38] and levels of transthyretin [39]. In addition, emerging evidence [10, 33, 34, 38, 39] and recent guidelines [40] are supportive of progressive provision of energy and protein during the first week of ICU admission.

## Conclusions

By integrating the predictors such as baseline nutritional status and disease severity, the GLIMPSE model demonstrated good prognostic performance in the prediction of 28-day mortality in critically ill patients. However, these predictors did not modify the association between the dose of nutrition support during the first 6 days of ENS and 28-day mortality. This suggests that the mortality-modifying effects of nutritional support are independent of the baseline nutritional status and disease severity of critically ill patients.

## Additional file

Additional file 1: Supplementary data. (DOCX 14 kb)

## Data Availability

The datasets generated and/or analysed during the current study are not publicly available due to the data confidentiality requirements of the ethics committee; however, they are available from the corresponding author on reasonable request and approval from the ethics committee.

## Abbreviations

APACHE II: Acute Physiology and Chronic Health Evaluation II
ENS: Exclusive nutritional support
GLIMPSE: Global Index of Mortality Probability in the Severely ill
ICU: Intensive care unit
mNUTRIC: Modified Nutrition Risk in Critically Ill score
SGA: Subjective Global Assessment
SOFA: Sequential Organ Failure Assessment
