# Supplementary material for: The association between nutritional adequacy and 28-day mortality in the critically ill is not modified by their baseline nutritional status and disease severity
Source: Crit Care. 2019 Jun 18;23:222. doi: 10.1186/s13054-019-2500-z (PMC6580600; doi:10.1186/s13054-019-2500-z)
Supplement: Supplementary file 1 — Supplementary data. (DOCX 14 kb) [file 13054_2019_2500_MOESM1_ESM.docx]

**Supplementary Data**

1. Beta weights (95% CI) and tolerance values of predictors as well as the Homer-Lemeshow Goodness-of-Fit of the GLIMPSE model:

- Tolerance values of predictors (generated by multiple linear regression):
  - Seven-point SGA: 0.9469
  - mNUTRIC score: 0.9261
  - Exposure to cardiopulmonary resuscitation before ICU admission: 0.9772
- Beta and 95% confidence intervals of predictors:
  - Seven-point SGA: -0.1661 (95%CI: -0.3319, -0.0002)
  - mNUTRIC score: 0.4768 (95%CI: 0.3309, 0.6227)
  - Exposure to cardiopulmonary resuscitation before ICU admission: 1.9327 (95%CI: 1.2342, 2.6312)
  - Constant: -3.0025 (95%CI: -4.4145, -1.5905)
- Hosmer-Lemeshow Goodness-of-Fit of our model:
  - 9.97, p-value 0.2670

1. Beta weights (95% CI) of potential confounders (i.e. *location before ICU admission*, and *types of admission*) that were not included in the multivariable logistic regression in the validation group

- The following are the results of the adjustment of *location before ICU admission*, *types of admission*, *GLIMPSE group*, *adequacy of energy intake* and *number days on exclusive nutrition support* in a multivariable logistic model
  - Location before ICU admission: 0.5606 (95% CI: -0.1996, 1.3209), p-value 0.148.
  - Types of admission: -0.0725 (95% CI: -0.7855, 0.6406), p-value: 0.842.
  - GLIMPSE group: 2.2751 (95% CI: 1.4493, 3.1009), p-value < 0.001
  - Adequacy of energy intake: 0.0205 (95% CI: 0.0043, 0.0367), p-value: 0.013
  - Number days on exclusive nutrition support -0.1325 (95% CI: -0.2153, -0.0497), p-value: 0.002
  - Hosmer-Lemeshow Goodness-of-Fit: 11.00 p-value: 0.2017
- The following are the results of the adjustment of *location before ICU admission*, *types of admission*, *GLIMPSE group*, *adequacy of protein intake* and *number days on exclusive nutrition support* in a multivariable logistic model
  - Location before ICU admission: 0.5818 (95% CI: -.1705, 1.3341), p-value 0.130.
  - Types of admission: -0.1304 (95% CI: -0.8361, 0.5753), p-value: 0.717.
  - GLIMPSE group: 2.2865 (95% CI: 1.4667, 3.1064), p-value < 0.001
  - Adequacy of protein intake: 0.0105 (95% CI: -0.0038, 0.0247), p-value: 0.150
  - Number days on exclusive nutrition support -0.1063 (95% CI: -0.1889, -.0238), p-value: 0.012
  - Hosmer-Lemeshow Goodness-of-Fit: 17.96 p-value: 0.0215
  - Omitting *location before ICU admission* and *types of admission* resulted in a Hosmer-Lemeshow Goodness-of-Fit of: 8.78, p-value: 0.3610.
